# Similar Conditions With Opposite Effects: Predation‐Risk Effects on Prey Abundance Are Highly Contingent

**DOI:** 10.1002/ece3.70861

**Published:** 2025-01-15

**Authors:** Scott D. Peacor, Clayton E. Cressler, Kevin L. Pangle, Alexandra V. Rafalski, Chao Song, Earl E. Werner

**Affiliations:** ^1^ Department of Fisheries and Wildlife Michigan State University East Lansing Michigan USA; ^2^ School of Biological Sciences University of Nebraska Lincoln Nebraska USA; ^3^ Department of Biology and Institute of Great Lakes Research Central Michigan University Mount Pleasant Michigan USA; ^4^ State Key Laboratory of Herbage Improvement and Grassland Agro‐Ecosystems, and College of Ecology Lanzhou University Lanzhou Gansu China; ^5^ Department of Ecology and Evolutionary Biology University of Michigan Ann Arbor Michigan USA

**Keywords:** context dependent, ecology of fear, *Lepomis*, nonconsumptive effect, nonlethal effect, trait‐mediated

## Abstract

Experiments have shown that predation‐risk effects on prey fitness can be highly contingent on environmental conditions, suggesting a potential difficulty in generalizing risk effects on prey abundance in natural settings. Rather than study the influence of a particular controlled factor, we examine the problem with a novel approach. We examined the influence of risk effects in multiple experiments performed under similar study conditions. Any differences in the experiments would typically be deemed incidental, that is, they would not be given attention in methodology, nor be presented as factors affecting results or inferences. Therefore, any differences in the magnitude and direction of risk effects among experiments would indicate that risk effects on prey population abundance are strongly influenced by context in natural communities. The multiple experiments were conducted under similar conditions, objectives, measurables and implementation, and captured much of the complexity of natural systems (e.g., they were performed with diverse prey assemblages (≥ 11 taxa) over multiple prey generations). Our results highlight the potentially profound context dependence of risk effects: risk effects on the density of some zooplankton species varied between a significant negative effect in one experiment to a significant positive effect in another, whereas other species showed significant negative or positive effects in one experiment and no effect in another. We review mechanisms that could underlie risk effects having opposite effects on the same prey. Our findings illustrate that risk effects observed in one study may not hold, even for the same species in the same system.

## Graphical abstract

Experiments in simple systems have shown that predation‐risk effects on prey fitness can be highly contingent on environmental conditions, suggesting a potential difficulty in generalizing risk effects on prey abundance in natural settings. Here we show that the response of zooplankton communities, and even the density responses of component species, to the presence of fish predators can vary from positive, to no effect, to negative across experiments that have identical set ups. Our findings highlight the need to understand the mechanisms linking trait responses to fitness and, ultimately, to abundance of prey, to understand risk effects across studies and systems.

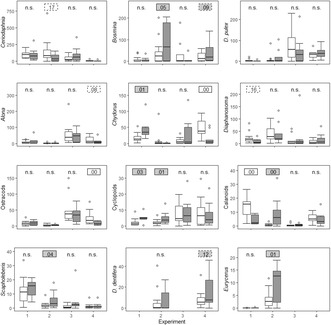

## 1 Introduction

The presence of predators often causes prey to modify traits, including behavior, physiology, morphology, and life history, in order to reduce predation risk (Lima and Dill 1990; Lima 1998a; Agrawal 2001). Such risk‐induced trait responses and their ensuing consequences, termed risk effects (also nonlethal effects and fear effects; terminology reviewed in Peacor et al. 2020), have received much attention. Whereas risk‐induced trait response in prey generally benefit prey by reducing predation risk, they also may have associated costs to fitness components in the prey. These consequences of the trait response on prey are termed nonconsumptive effects (NCEs), and include reduced growth or condition, reduced fecundity, or increased mortality from other predators, and consequent effects on prey population growth rate and prey abundance (Abrams 1984; Brown 1988; Lima 1998b; Peckarsky et al. 2008; Ohgushi, Schmitz, and Holt 2012; Sheriff et al. 2020; Peacor et al. 2022). The risk‐induced trait‐responses of the prey can also have indirect effects (termed trait‐mediated indirect effects, TMIEs; Peacor et al. 2020), with other species including their resources, competitors, or other predators (Sih, Enlund, and Wooster 1998; Werner and Peacor 2003). NCEs and TMIEs have received much attention given their potential to profoundly influence ecological communities and management of natural resources (reviewed in Werner and Peacor 2003; Schmitz, Krivan, and Ovadia 2004; Creel and Christianson 2008; Heithaus et al. 2008; Peckarsky et al. 2008; Ohgushi, Schmitz, and Holt 2012; Say‐Sallaz et al. 2019).

Indeed, it has been argued that risk effects are as important to the net effect of predators on prey abundance as their direct consumptive effects (reviewed in Sheriff et al. 2020). However, that conclusion has been based largely on experiments done in relatively simple systems with only a few interacting species and little temporal or spatial environmental heterogeneity (Nelson 2007; Sheriff et al. 2020), even when performed in natural settings (Sheriff et al. 2020; Peacor et al. 2022). Thus, if we are to incorporate risk effects into ecological models and management plans, it is important to know that the effects demonstrated in simplified systems are generalizable to more complex systems incorporating many interacting species and spatial and temporal heterogeneity, and how generalizable findings in natural systems are across time and to other similar systems.

Here we investigate the potential generalizability of risk effects with a novel approach. We examine the risk effect of fish on the density of different zooplankton prey species in complex experimental communities with multiple species over multiple generations, thus allowing for the type of indirect effects and feedback expected in natural systems. The novelty of our approach is that we look at risk effects across multiple experiments, each with a very similar experimental conditions, objectives, measurables and implementation. Thus, any observed variability in the effect of predation risk across experiments arises from differences in the experiments that are purely incidental. By “incidental,” we mean that these were differences that, based on existing studies and our own experience, would not typically be viewed as influencing study outcomes, for example, they would not be given attention in methodology, nor be presented as factors affecting results or inferences. Our approach is thus different than most studies that have identified context dependence in the effect of ecological factors on fitness components over short time scales. Typically, a particular biotic factor, such as prey density, or abiotic factor, such as physical complexity from plants, is systematically varied to investigate the influence of that factor. We ask, rather, could factors that are typically unmentioned and unreported (because they are deemed inconsequential by the investigators) influence the results of a study of risk effects in a natural system? Given that experimental systems are necessarily simplified, compared to the full complexity of natural systems, our approach represents a conservative examination of contingency in natural settings. We find that, even in simplified systems, incidental variation can have a large influence on NCEs and even alter the sign of risk effects, suggesting that in natural systems risk effects may be highly context dependent, and caution must be taken to generalize results or risk effect studies.

## 2 Methods

### 2.1 General Approach

We compared the risk effects of fish on the density of multiple zooplankton taxa across four experiments performed in different years, but with similar conditions, objectives, measurables and implementation. The experiments all included shared treatments (among others) that allow us to examine fish risk effects on zooplankton density by comparing caged‐fish and no‐fish treatments. The NCE on the density of given zooplankton taxa could thus be compared across experiments to identify the contingency of risk effects. Our approach is novel in that we ask: do experiments set up to test for the same risk effect in a complex community yield the same results qualitatively? To be clear, we had no a priori prediction of whether differences among experiments would drive differences in the magnitude or direction of risk effects large enough to be observed.

The venue was constructed to capture much of the complexity of natural systems. Each experiment used the same large mesocosms set outdoors (subject to natural environmental variation) and used a natural resource base (phytoplankton) supported by nutrient addition, and a speciose (> 10 taxa) zooplankton prey assemblage. Each experiment ran for 3–5 generations of the prey species with the longest generation times (see Peacor et al. 2012), allowing the potential for ecological feedbacks to affect density. Although the venue is obviously less complex than a natural system (e.g., lacking in other predator taxa), it incorporates much more of the complexity of a natural system than a typical laboratory experiment that has few prey species, a simple and controlled resource base, and controlled abiotic conditions.

We provide a description of the experimental methodology that was common to all experiments. Where there were differences among experiments, such as small differences in the average size of fish used, a range is provided. Wherever a range is provided in the methods the specific details are provided in a table in the Supporting Information. Other differences among experiments and methodological differences are summarized after the general description and described in more detail in Supporting Information. The timing of manipulations and samplings are given relative to the day experimental treatments were initiated (as provided in Supporting Information), defined as day 0. The key point is that all of these differences are incidental—in any paper examining risk effects, whether in experimental containers or the field, these factors would not be considered important in methodology (e.g., there would be no control for them) and would not be mentioned as potentially driving the inferences made.

### 2.2 Methodology Shared Among Experiments

Outdoor mesocosm experiments were conducted at the E. S. George Reserve (ESGR) of the University of Michigan near Pinckney, Michigan, USA (42°28′ N, 84°00′ W). Cylindrical cattle watering tanks were employed and contained approximately 1100 L of well water. Tanks were 1.9 m in diameter and 0.75 m tall, and were filled to a depth of 45 cm. Washed sand was added to each mesocosm as a bottom substrate. Each mesocosm was covered with a fiberglass window screen lid to deter colonization by insects. On particularly sunny days, 60% green shade cloth lids were used on top of the window screen lids to reduce heating.

The design of each experiment included the presence/absence of fish kairomone (i.e., chemicals) and additional treatments to explore different questions in each experiment (Supporting Information). A randomized block design was used for each experiment, and replicates ranged from 6 to 16 depending on the treatment and experiment. The nonconsumptive effect of fish was created by maintaining one zooplanktivorous bluegill sunfish, *Lepomis macrochirus* (standard lengths given in Supporting Information) in each of two or three floating cages within each kairomone treatment mesocosm. No‐fish treatments consisted of mesocosms with the same number of empty floating cages. Large holes in the sides and bottom of the boxes were covered with fine netting to allow for diffusion of fish kairomones without permitting zooplankton to pass through. In Experiments 1 and 2, three large snails (*Planorbella cf. trivolvis* > 11.2 mm in diameter) were kept inside each cage to graze on periphyton that could grow on the mesh windows. Adding snails was deemed extraneous and not implemented in Experiments 3 and 4.

Fish originated from Patterson Lake, Livingston County, Michigan. In order to ensure fish health and to equalize fish cue (e.g., in case fish differed in cue production) we rotated the fish from the experimental mesocosms to a culture tank once a week. Culture tanks consisted of outdoor mesocosms of ~50 fish of similar size fed zooplankton three times per week. Fish in culture tanks were not fed for 24 h before being rotated back into the experiment. Although in the experimental cages, each fish was fed twice a week, including the day they were added to the cages. To feed fish, an average of 200 *Daphnia* were added per cage. The no‐fish cages received an equal number of *Daphnia* that were killed by microwaving to ensure a population did not build in the cages.

Calculations of nutrient inputs indicate that nutrients from fish excretion were inconsequential, as they were overwhelmed by other sources, including external supply and internal recycling by zooplankton (See Supporting Information in Peacor et al. (2012)). Two experiments also provide evidence that nutrient inputs from fish had no ecological effects. In one, caged fish had no effect on phytoplankton growth in chambers placed outside, but near, the cages and performed soon after (8–10 days) treatments were initiated. In a second in situ experiment, fish excretion had no effect on phytoplankton growth in chambers placed within the fish cages (Rafalski and Peacor, unpublished).

An initial pulse of nutrients (see below) and a phytoplankton inoculum were added to each tank between 35 and 56 days before the start of the experiment. The phytoplankton inoculum consisted of water collected from ponds in the ESGR and filtered through 35 μm Nitex mesh. A community of zooplankton was added to each tank between day −49 and −27 (Experiment 3 received two inoculums). This initial inoculum came from a single lake (Experiments 2–4) or multiple lakes (Experiment 1, see Supporting Information for details on source lakes). We collected zooplankton using a 64 μm zooplankton net, and insects (e.g., *Chaoborus*) and *Hydra* were removed prior to adding the inoculum to the mesocosms. To decrease zooplankton heterogeneity among mesocosms, on day −24 to −6 we collected samples of zooplankton from each mesocosm using a 64 μm zooplankton net, mixed all samples, and redelivered subsamples of this mixed community to all mesocosms. The procedure was done twice for Experiments 3 and 4. Although this procedure reduces variation in initial densities among tanks, there will clearly be more variation at treatment initiation than in a more controlled experiment that would start with constant densities.

Inorganic nutrients were supplied to the mesocosms to support phytoplankton growth as a resource for zooplankton at an N:P ratio of 15:1 or 20:1 (Supporting Information). An initial pulse of NH_4_NO_3_ and KH_2_PO_4_ was added to each mesocosm when phytoplankton were added, and thereafter we supplied maintenance doses weekly. The rate and overall amount of nutrients added were similar among experiments (Supporting Information), though there was some variation in rates and the frequency at which nutrients were added, varying from twice per week to continuous (Supporting Information). In some cases, the weekly rate was reduced to reduce phytoplankton and filamentous algal growth when deemed excessive (Supporting Information). To reduce periphyton growth and to cycle nutrients back to the water column, we collected visible filamentous algae by hand each week, clumped it, allowed it to dry and returned it to the mesocosms. In Experiments 1 and 2, we also added *Planorbella cf. trivolvis* snails to each mesocosm to reduce periphyton growth.

Zooplankton were sampled between days 31 and 71. Samples were passed through a 53–64 μm mesh sieve and preserved. Zooplankton were identified and enumerated. Zooplankton identification was resolved to species or genus for Cladocera, adult cyclopoid and adult calanoid for Copepoda (referred to as “cyclopoid” and “calanoid”, exception described below), and ostracods for Ostracoda.

There were a number of methodological differences among experiments due to the specific questions addressed which we summarize here and describe in more detail in Supporting Information. (1) The zooplankton collection method differed; in Experiments 1 and 2 we subsampled different positions in the tank to determine both zooplankton position in the tanks and overall density, whereas in Experiments 3 and 4 we measured overall density from combined samples of the entire water column. This difference should not affect our assessment of risk effects of fish on zooplankton density, as the data we are reporting from Experiments 1 and 2 is pooled across different tank positions. (2) The experiments examined different aspects of risk effects with additional treatments. Experiments 1 and 2 included a treatment with an invertebrate predator (notonectids and *Chaoborus*, respectively). However, these treatments showed no differences in zooplankton density (based on generalized linear models) or community structure (based on PERMANOVAs) compared to the no‐fish treatment, so they were included in that treatment to increase power (Supporting Information B). Experiment 4 included a treatment where periphyton was removed from the tanks, crossed with fish and no‐fish treatments. Our manipulation had only a small effect on periphyton, and the periphyton manipulation had no effect on zooplankton density or community structure, so these tanks were pooled into the no‐fish or fish treatments. (3) The origin of the zooplankton was the same (a single lake) for Experiments 2–4, but differed for Experiment 1 in which collection was from multiple lakes. (4) *Hydra*, which are predators of some zooplankton species, were inadvertently present in Experiment 1 (presumably introduced through the zooplankton inoculum), but not Experiments 2–4. (5) Because the size and ecological role of the juvenile stage of cyclopoids can be variable (Burns and Gilbert 1993), we counted only adult cyclopoids. However, in Experiment 4 the counting resources were more limited and thus the cyclopoid counts include both juveniles and adults (i.e., they were not differentiated).

All procedures involving animals were approved by University of Michigan's Committee on the Use and Care of Animals under approval number 07765 (for Experiments 1 and 2) and Michigan State University's Institutional Animal Care and Use Committee approval no. 02/12–025‐00 (for Experiments 3 and 4).

### 2.3 Statistical Analysis

We compared the risk effect of fish on zooplankton densities across the four experiments for the subset of zooplankton taxa that were represented in all experiments (see Section 3, Table 1). We used a multivariate approach to evaluate the overall response of the zooplankton community to the presence of fish kairomones, and our initial analysis simultaneously tested the effect of experiment, treatment and the interaction between experiment and treatment on the subset of taxa using a permutational multivariate analysis of variance (PERMANOVA, McArdle and Anderson 2001). This analysis uses dissimilarity matrices, and we chose to use a dissimilarity matrix based on Kulczynski distances, which are particularly robust in identifying patterns in community data (Faith, Minchin, and Belbin 1987). Probabilities from the analysis were calculated based on 9999 permutations. These effects were also investigated graphically by comparing experiments and treatments across different axes generated through nonmetric multidimensional scaling (NMDS). The NMDS was based on Kulczynski distances and was constrained to three dimensions, which was the minimum number of dimensions that resulted in a stress measure < 0.20 (Clarke 1993).

**TABLE 1:** Top box: Zooplankton mean density (/liter) and standard deviation across four experiments, ordered by most to least common from left to right.

	*Ceriodaphnia*	*Bosmina*	*D. pulex*	*Alona*	*Chydorus*	*Diaphanosoma*	Ostracods	Cyclopoids	Calanoids	*Scapholeberis*	*D. dentifera*	*Eurycerus*	*Simocephalus*	*D. parvula*	*Pleuroxis*
Mean density	73	55	29	27	23	22	18	5.8	5.1	5.0	4.4	1.4	0.8	0.7	0.5
SD density	49	68	27	36	12	12	19	2.3	4.2	4.9	5.6	2.8	0.6	1.5	0.6
Ranks															
Experiment 1	1	8	***11***	5	2	3	7	9	6	4	N	13*	***12***	10*	N
Experiment 2	2	1	4	***14***	***7***	3	11	8	9	10	5	6	13*	N	12
Experiment 3	2	***6***	3	1	5	***7***	4	8	***10***	***9***	N	N	11*	N	N
Experiment 4	9	2	1	4	3	***7***	5	8	10	11	6	N	N	N	12*
Effects															
No, Pos., Neg.					**X**				**X**						
No, Neg.				**x**			**X**								
No, Pos.		**X**						**X**		**X**					
All No	**X**		**X**	**X**		**X**									

*Note:* *Daphnia retrocurva* and *Macrothrix* are not included as they were at very low densities in a small number of tanks of Experiment 1 only. Middle box: Density rank of each taxa in each experiment, and the percent of tanks the taxon was found in as indicated by: Plain text for 90%–100% occupied, italicized bold text for 70%–90% occupied, underlined text for 50%–70% occupied, an asterisk for 20%–50% occupied, and N for no tanks occupied. Bottom box: The caged‐fish treatment effect combinations of no effect, positive effect or negative effect, seen among experiments (see Figure 1) is given by a capital X for each of the 10 taxa found in all four experiments. The lower‐case x represents the combination trend if *p* < 0.1 is used rather than *p* < 0.05.

The effect of treatment on the density of individual taxa within an experiment was analyzed using quasi‐Poisson regressions. The quasi‐Poisson distribution was appropriate here, as it provides a better fit to count data, especially when many of the measurements are close to zero (Ver Hoef and Boveng 2007).

## 3 Results

**FIGURE 1:**
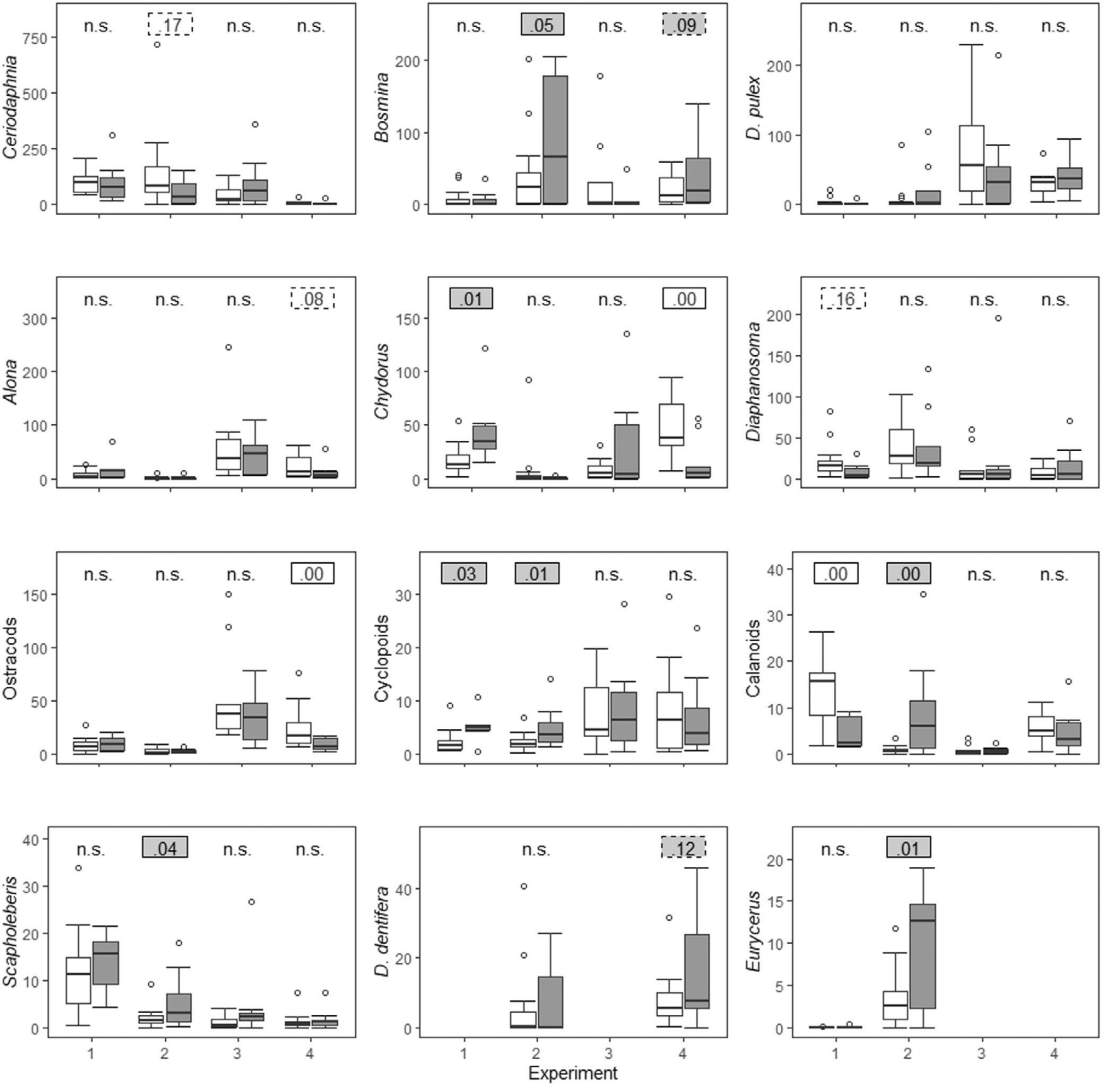
Whisker plots of the density (# ind/l) of the 12 most common zooplankton taxa in the four experiments. Significant effects are indicated with *p*‐values (in solid outlined boxes). Where a strong trend is evident in the figure, *p* values are also provided (in dashed outlined boxes), and n.s. signifies *p* > 0.2. Filled and empty boxes represent positive and negative effect of the caged predator, respectively Taxa given in order (right to left, top to bottom) of average density (Table 1). The dark line in the boxes is the median, and the bottom and top of the box is the 25th (Q1) and 75th percentile (Q3) of the data. The lower whisker is Q1–1.5*IQR (the interquartile range, between the 25th and 75th percentile), and the upper whisker is Q3 + 1.5*IQR. Points are outliers, and the following density values were removed from the figure to better illustrate the core distribution of densities: 2400 for *Bosmina* in Experiment 2, Fish Treatment; 238.0 for *Chydorus* in Experiment 3, Control Treatment; 43.8 for cyclopoids in Experiment 2, Fish Treatment; and 505.5 for *Alona* in Experiment 3, Fish Treatment.

**FIGURE 2:**
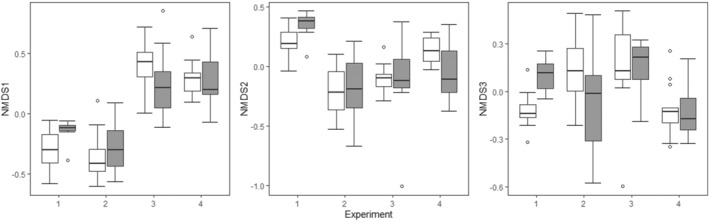
NMDS values for each of the four experiments in the caged‐fish and no‐fish treatments. Whisker plot as in Figure 2.

Zooplankton species richness was similar in all four experiments, with the 10 taxa that had the highest average density across experiments found in all experiments (Table 1). Upon visual inspection of the data, the mean and standard deviation of the density of taxa varied greatly among experiments (Figure 1, Table 1). The density ranks also varied among experiments, for example, the most common (Rank 1) species in one experiment could be anything from the 2nd to the 14th most common taxa in another experiment. Nevertheless, there were some strong similarities among experiments. For example, the rank of some taxa (*Chydorus*, cyclopoids and calanoids) did not vary by more than 4. Further, *Ceriodaphnia* was the first or second most common species in Experiments 1–3 (but much less common in Experiment 4 in which *Daphnia* and *Bosmina* were particularly common). We also report occupancy of each taxon as the percentage of tanks in which it was found. Only in one case was the occupancy of a common taxon (i.e., found in all four experiments) < 70% (in Experiment 1, *Bosmina* was found in 54% of the tanks). Variation in community composition of zooplankton across experiments was much larger than the variation within an experiment, as shown by the significant effects of experiment (*R* ^2^ = 0.327, *p* = 0.0001) in the permutation multivariate analysis (PERMANOVA), and illustrated in Figure 2, in which the NMDS values differ among experiments.

Caged fish had an effect on zooplankton community composition that varied across experiments, as evidenced by the significant experiment × caged fish interaction in the PERMANOVA (*R* ^2^ = 0.050, *p* = 0.0036), but no effect of caged fish when averaged across the four experiments (*R* ^2^ = 0.007, *p* = 0.47). This varying effect of caged fish on community composition is evident in Figure 2, as the NMDS values of the caged‐fish treatment were observed to be higher, lower, or not different than the values of the no‐fish treatment in different experiments (e.g., for NMDS dimension 3, the fish treatment is higher than the no‐fish treatment in Experiment 1, but lower than the no‐fish treatment in Experiment 4). In the Supporting Information, we show scatterplots of each community along the NMDS axes, with ellipses showing how communities are clustered within treatments and experiments.

Caged fish had a significant effect on the density of some taxa, and in some cases the direction of that effect varied among experiments (bottom box, Table 1). For two taxa all three possible effects occurred: the presence of caged fish increased, decreased, and had no effect on the density of calanoids and *Chydorus*. For four taxa two possible effects occurred: fish increased or did not affect the density cyclopoids, *Bosmina* and *Scapholeberis*; fish decreased or did not affect the density of ostracods. There were also several cases where there was a significant positive or negative effect of fish on a taxon in one experiment, and a nonsignificant trend in the opposite direction in another experiment. For example, there is a significant negative fish effect in Experiment 4 on ostracods, and the “no effect” result in Experiment 1 shows a trend in the positive direction with small enough within‐treatment variation that the influence is unlikely in the negative direction. In all cases in which there was a positive or negative effect of fish, the taxa were observed in all tanks in both treatments. For four taxa fish did not have a significant effect in any of the experiments (*Alona*, *Ceriodaphnia*, *D. pulex* , and *Diaphanosoma*).

## 4 Discussion

The principal result of this study is that the effect of predation risk on the density of a given prey taxon varied significantly across experiments conducted under similar conditions, objectives, measurables and implementation; the effect of caged fish on the density of the same taxa ranged among experiments from negative or no effect, positive or no effect, and negative, positive or no effect. A multivariate analysis similarly indicated an influence of caged fish on the zooplankton community composition that varied among experiments. Our approach is conservative in nature, as the variability introduced by the incidental differences in experimental design across the four experiments (e.g., the date of phytoplankton inoculation or the presence of snails) is expected to be much larger in nature than in our experiments. Our study indicates that if a predator is found to have a positive or negative risk effect on prey abundance in a particular system, or at a given time, this alone may yield limited information on the existence, magnitude or direction of a risk effect on prey abundance in another location or time.

Contingency in risk effects, as found in our study and generally, is complex as it can arise at any of three scales (Sheriff et al. 2020; Wirsing et al. 2020): a predator induces changes in prey phenotype (e.g., behavior, morphology), which can lead to changes in prey fitness components (e.g., fecundity, survival), which in turn can influence prey population growth rate and abundance. For the first effect, empirical studies have shown that the magnitude, and even existence, of a trait response is contingent on the balance of costs and benefits associated with the trait change, each of which is strongly dependent on biotic and abiotic factors, such as resource level, the presence of other predators, con‐ and heterospecific density, and abiotic factors such as temperature and landscape features (Thaker et al. 2011; Tollrian et al. 2015; Donelan, Grabowski, and Trussell 2017; Wirsing et al. 2020). For example, prey are predicted to show a lower response at lower resource levels, which can be affected by many factors including competitors.

The second effect of a trait response on fitness components (i.e., NCEs) has also been shown to be strongly contingent on abiotic and biotic factors. For example, the cost of a habitat shift (the trait response) will depend on consequent experienced changes in temperature, resources, and mortality risk from other causes (e.g., parasites or other predators). Further, it has been shown that the level of trait responses of intraspecific or interspecific competitors to predators can cause the NCE on a focal prey's fitness to be magnified, dampened and even reversed (Relyea 2000; Peacor and Werner 2004).

Previous studies that intentionally manipulate aspects of the abiotic or biotic environment thus clearly show contingency in risk effects on both trait and fitness components, and altering either response could alter predation‐risk effects on prey abundance. Indeed, theoretical studies show very clearly that altering responses “upstream” of prey abundance can alter abundance responses (Abrams and Vos 2003; Bolker et al. 2003; Peacor and Cressler 2012). However, there are very few empirical studies that show contingency in risk effects on prey abundance (for an example see Baker et al. (2022), reviewed in Sheriff et al. (2020)). Moreover, there was high within‐treatment replicate variability compared to typical NCE experiments. For example, different replicate mesocosms within an experiment often varied in characteristics across experiments such as periphyton growth and zooplankton taxa density (density coefficient of variation equal to 1.2 ± 0.62 [mean ± SD] within treatments for all taxa across the four experiments). The high levels of variability observed within treatments, despite the relatively simplicity of the environment, has two important implications. First, high variation within treatments would make significant risk effects less likely to be observed, as any effect of the caged fish on prey density had to be large to be statistically detectable over the high within‐replicate variation. Second, the fact that we observed contingency despite the high similarity among experiments relative to differences among natural systems, and despite the high within treatment variation relative to typical experiments of risk effects, suggests that this study provides a conservative test of the contingency of risk effects on prey abundance in natural settings.

Nearly all of the important species in our experimental communities have been reported to be components of bluegill diet in lakes and ponds. In particular, *Daphnia*, *Ceriodaphnia*, *Bosmina*, *Chydorus*, copepods, ostracods, and *Simocephalus* can at times be a dominant or substantial component of bluegill diets (e.g., Hall, Cooper, and Werner 1970; Keast 1985; Rettig and Mittelbach 2002; Dewey, Richardson, and Zigler 1997; Bremigan and Stein 1994). Consequently, these species have an evolutionary history of risk to bluegill predation, and indeed, many have been shown to respond phenotypically to presence of fish, including in some cases bluegill, and to other predators. For example, a large number of studies have documented phenotypic response in *Daphnia* to a wide array of predators including bluegill (see reviews in Lass and Spaak (2003) and Diel et al. (2020)). Diel et al. (2020) also report evidence of phenotypic responses to predators in *Bosmina* (including to fish), *Diaphanosoma*, *Simocephalus* (including to fish), *Ceriodaphnia* (including to fish), copepods, and *Chydorus* (to injured conspecifics, Pecor et al. 2016). In experiments of the same venue used here, we have further observed fish kairomones to affect the spatial position of *Alona*, *Bosmina*, *Chydorus*, *Diaphanosoma*, *Scapholeberis* and ostracods (unpublished data). Thus, many of the important components of the zooplankton communities in the experiments have the potential to respond to the presence of bluegill kairomones and initiate effects we see of bluegill presence on community structure.

Perhaps surprisingly, there was no risk effect of caged fish on the density of some vulnerable zooplankton prey in some or multiple experiments. For example, using bluegill of the same size and origin, we found that bluegill preference (as measured by Chesson α selectivity index) was highest for *D. pulex* and *Ceriodaphnia* (Rafalski, Pangle, and Peacor 2023) which is consistent with other studies that report preferred prey of small bluegill (Bremigan and Stein 1994). Yet, there was no NCE on *D. pulex* or *Ceriodaphnia* density in any of the four experiments, (though there is a nearly significant negative trend in Experiment 2 on *Ceriodaphnia*). Moreover, other mesocosm studies have found NCEs of fish on these species (e.g., Baker et al. 2022). The lack of an effect cannot be explained by the absence of a cue of predation risk in the experimental venue (e.g., kairomones can be affected by properties of the water (Peacor 2006, Turner and Chislock 2010)) because other species were affected by caged fish in Experiments 1, 2 and 4, whereas in Experiment 3 multiple species responded behaviorally to caged fish (Rafalski et al. unpublished). A power analysis confirmed that the lack of any significant effects in Experiment 3 was not due to a lower sample size or higher variance in that experiment, but rather to a true lack of effect of fish on the densities of any species (Supporting Information). Our results therefore highlight that NCEs can, depending on context, be much weaker than CEs such that predators may have no NCEs on the density of vulnerable prey in some circumstances (Hoverman and Relyea 2012). It is beyond the scope of this study to identify the specific factors responsible for the observed contingency in NCEs in our study, but we can speculate. Incidental differences (as defined here and not to be interpreted as uninfluential effects, see Methods) in factors such as temperature and initial relative abundances could lead to variation in community assembly processes across experiments (Drake 1991; Chase 2003) that could lead to the differences in species densities observed among experiments, and those differences in species densities could influence predation‐risk effects. For example, in Experiment 2 *Ceriodaphnia* density was higher in the no‐fish treatment than it was in the other experiments, and it was the most abundant zooplankton taxon (Table 1, Figure 2). We have found that *Ceriodaphnia* responds behaviorally to caged bluegill by moving horizontally away from the tank center (unpublished data) that would likely be associated with reduced foraging rates or freeing of resources in the center of the tanks. The indirect benefit to other zooplankton taxa due to competitive release would increase as a function of *Ceriodaphnia* density, and thus would be most likely observed in Experiment 2 where indeed positive effects were observed on a number of taxa. There was a nearly significant trend for predation risk to reduce *Ceriodaphnia* density in Experiment 2, which could have further contributed to the observed positive effects on other species densities. Additionally, the length of the experiments (relative to generation times; even the species with the longest generation time experienced at least three generations; Peacor et al. 2012), allowed ample time for incidental differences among experiments to generate ecological feedbacks, for example, generating indirect effects on resources. Indirect positive effects on resources could also vary among experiments because of differences in species densities among experiments, or a predator‐induced reduction in foraging on particular phytoplankton species that facilitated growth of other phytoplankton species favored by different zooplankton species.

The presence of *Hydra* in Experiment 1 is another factor that could affect how caged fish influence zooplankton and hence cause contingency. If caged fish induce a habitat shift in zooplankton to be closer or further from surfaces where *Hydra* reside, this would make them more or less vulnerable to *Hydra* predation, respectively. Ensuing effects on density of one zooplankton species could then have indirect effects on the density of other zooplankton species. Note that effects of *Hydra* cannot be responsible for all of the contingencies observed, as different effects of caged fish remain if Experiment 1 is omitted (top box, Table 1).

It is also possible that some contingency is manifested because of species composition differences in broader taxonomic groups in our study (e.g., cyclopoids and calanoids) across experiments (and such species reacted differently to the bluegills). This possibility, however, does not apply to more circumscribed categories, for example there is only one species of *Chydorus* in the region ( *Chydorus sphaericus* ), and caged fish had a positive, neutral, and negative effect on *Chydorus* density.

More generally, the change in effects across experiments could be a manifestation of higher‐order effects (Abrams 1983; Kleinhesselink et al. 2022) or interaction modifications (Wootton 1993, 1994), whereby differences in the abundance of one species influences the interaction of two other species, which can affect the fitness and abundance of a those species, and so on, rippling through the community of ecological interactions to amplify the initial differences in species one abundance (Levine et al. 2017). Thus, any factor (including those summarized here) that affects the traits or fitness of one species that consequently influences their abundance could contribute to the contingency of predation risk on other species.

Overall, there were many differences between the four experiments analyzed here. For some of these factors, we can speculate (as we do in the preceding paragraphs) as to how they might be the driver of the observed differences among experiments. But there are myriad other factors that we either cannot imagine a mechanism for their effect, or we did not even measure them. Clearly one of these factors, either by itself or through an interaction with other factors, underlies the differences we observed between experiments in the response of prey density to predation risk. Thus, though these factors might be deemed as incidental because they would not be referenced as influencing the results of the experiment, they were clearly consequential. We are thus not arguing that such uncontrolled or unmeasured factors are inconsequential. Nor are we arguing that field studies of NCEs will require greater levels of control to isolate these factors; to some extent that will always be impossible, for example, because of differences in weather between years. Weather is a good example of a factor that is typically deemed incidental; although interannual variation is common, few field experiments are repeated across multiple years to ensure that the effect observed in 1 year is repeated; that implicitly reflects a belief that factors like the weather are incidental to the main inferences of the study. Rather, our point is that the contingency observed across the experiments elucidates the strong contextual nature of NCEs on population abundance. As active researchers in the theory and empirical effect of risk effect, we were aware of the possibility, but did not foresee, that the uncontrolled or unmeasured factors that varied among these experiments would completely reverse the direction of NCEs for some prey species, and yet they did.

It is important to establish the degree to which risk effects on prey abundance are contingent for at least two reasons. First, contingency will make it challenging to translate findings of strong risk effects in simple experimental settings to natural communities or translate strong trait‐responses of prey seen in the field to a consistent prediction of ensuing effects on prey fitness and abundance. Second, much of the literature on risk effects has addressed whether they are strong relative to consumptive effects (CEs). For example, empirical (Werner and Peacor 2006) and meta‐analysis studies (Preisser, Bolnick, and Benard 2005) have compared the influence of risk effects and CEs, with an implicit assumption that they contribute different relative fractions of the net effect of a predator. But if risk effects on prey abundance are strongly contingent, then it is more difficult to make inferences from single reports of the magnitude of risk effects. If contingencies of risk effects on prey abundance are important, this highlights the need to understand the mechanisms underlying risk effects, and how environmental factors will influence the consequences of risk effects. Resolving such questions has clear implications for ecological theory and attempts to include the effects of risk effects into conservation and management, and future studies should attempt to compare the magnitude and reproducibility of NCEs relative to CEs.

We have demonstrated that predation‐risk effects on abundance of prey species can be highly contingent on conditions, varying even in sign, using methodology that likely underestimates the influence of contingency in natural systems. Many investigators state that risk effects are known to be important, implicitly implying that risk effects influence the abundance of species in natural systems (Sheriff et al. 2020). But few studies, in very few ecological systems, have actually tested for such effects on abundance in natural systems (Peacor et al. 2022), and until such tests are performed, our results on the contingency of risk effects suggest that results of an experimental study may not translate to a natural system (i.e., effects may vary in the two systems). An understanding of the underlying mechanism of any observed NCEs could assist in generalizing the effects. For example, if the NCE results from a reduction in foraging and energy gain, then factors that influence such gain (e.g., resource replenishment rates, inter‐ and intraspecific competition, Peacor and Werner 2004) could assist in predicting the influence of an NCE in another system if such factors are known. Nevertheless, many other factors may be at play, and our results point to the need for caution in generalizing results, either on the magnitude or even the sign of NCEs. This may be particularly important, given that theoretical studies have argued that risk effects have been exaggerated by omitting crucial factors such as stage structure that can decrease the influence of risk effects (Luttbeg, Rowe, and Mangel 2003; Persson and De Roos 2003), or by methodological problems used in experimental studies (Abrams 2010). Given the ubiquity of the mechanisms underlying risk effects (e.g., pervasiveness of phenotypic responses to predators), we believe that they likely play an important role in the dynamics and structure of ecological systems, but much work remains to better understand their role.

## Author Contributions

**Scott D. Peacor:** conceptualization (lead), data curation (lead), formal analysis (equal), funding acquisition (lead), investigation (equal), methodology (equal), project administration (lead), supervision (lead), visualization (equal), writing – original draft (lead), writing – review and editing (equal). **Clayton E. Cressler:** conceptualization (supporting), formal analysis (equal), investigation (equal), visualization (equal), writing – original draft (supporting), writing – review and editing (equal). **Kevin L. Pangle:** conceptualization (supporting), formal analysis (equal), investigation (equal), writing – review and editing (equal). **Alexandra V. Rafalski:** conceptualization (supporting), investigation (equal), writing – review and editing (equal). **Chao Song:** formal analysis (equal), methodology (equal), visualization (equal), writing – review and editing (equal). **Earl E. Werner:** conceptualization (equal), funding acquisition (equal), methodology (equal), writing – review and editing (equal).

## Conflicts of Interest

The authors declare no conflicts of interest.

## Supporting information


Data S1.

## Data Availability

Data and analysis scripts for this manuscript are archived on GitHub at https://github.com/claycressler/ZooplanktonCTs.
