# Supplementary material for: Similar Conditions With Opposite Effects: Predation‐Risk Effects on Prey Abundance Are Highly Contingent
Source: Ecol Evol. 2025 Jan 15;15(1):e70861. doi: 10.1002/ece3.70861 (PMC11735265; doi:10.1002/ece3.70861)

# Title: Similar conditions with opposite effects: Predation-risk effects on prey abundance are highly contingent

# Supporting Information A: Differences among experiments

## We outline differences between experiments that were not explained in the main text.

## Zooplankton collection method for density estimation

The zooplankton collection method for density estimation at the end of the experiment varied among experiments. In Exp. 1 zooplankton were collected from three strata: ‘high’ (just below the surface), ‘middle’ (at the midwater level) and ‘low’ (right above the bottom). Six samples were collected at each stratum with a 15 cm long 1.2 l horizontal water sampler (Wildco), four by the walls and two near the center of the tank and combined. The average density of the tank was defined as the average density across the three strata. In Exp. 2, the collection method was similar, except that there were 3 samples collected near the center rather than 2, and the center and side samples were not combined together (making a total of 6, rather than 3, samples per tank). The average density of the tank was defined as the average of the density across the six positions. In Exp. 1 and 2, zooplankton were sampled during the day, because the data was also used to explore whether cues from the caged fish affected zooplankton position, which is predicted to be affected during the day by the visual predator. In Exp. 3 and 4, a 5 cm diameter vertical tube sampler was used to collect samples of the entire water column (i.e., from the surface to the bottom of the mesocosm). Twenty tube casts were taken at points along a grid placed over the tanks and combined to yield one 18 L sample (~2% of mesocosm volume). Sampling was stratified along the radius of the tank such that the sampling near the middle and walls was proportional to their areas. Zooplankton were sampled after sunset because we observed less spatial segregation and thus presumed there would be more accurate sampling at night. Whereas it is possible that the methodology used in the different experiments varied in their ability to precisely estimate average tank density, all collection methods were thorough and represented a large and even portion of the tank; we therefore do not believe that the collection method would influence the estimate of the effect of fish on zooplankton density.

## Additional treatments

Exp. 1 and Exp. 2 each had a treatment with a second caged predator: notonectids and midges (*Chaoborus*), respectively. Compared to the no-fish treatment, these invertebrates did not influence zooplankton abundance or community composition (Supporting Information B) and thus we have included these replicates in the current analysis by combining them with the no-fish treatment to increase power. Exp. 3 used a 2 x 2 factorial design, with caged fish and a zooplankton removal treatment to simulate the consumptive effect of predators (as in e.g. Peacor and Werner 2001). Because the removal treatment was designed to directly affect zooplankton density, which it did, the removal treatments are not included in this analysis. Exp. 4 also used a 2 x 2 factorial design, with no fish and caged fish crossed with no manipulation and manipulation of periphyton growth on tank walls. We manipulated periphyton growth by lightly wiping tank walls with a scouring pad. Other treatments received the same motion in the tanks but without wiping the sides. The manipulation had a negligible effect on zooplankton density ( Supporting Information), likely in part because the manipulation had little effect on periphyton, and thus to increase power we combined the treatments that did and did not have the manipulation into the fish and no-fish treatments.

## Origin of zooplankton

The origin of the zooplankton differed in one of the experiments. In Exps. 2, 3 and 4, zooplankton were collected from a single small lake, Sayles Lake. Sayles Lake is a 10-hectare lake located on the E. S. George Reserve, with a maximum depth of approximately 3 m and a diverse fish community including bluegill. In Exp. 1, zooplankton were collected from four semi-permanent and permanent ponds and three small lakes within the E.S. George Reserve, including Sayles Lake. While there were several taxa only found in Exp. 1, such as *Macrothrix*, *Daphnia parvula*, and *Daphnia retrocurva*, these were rare, representing less than 0.2% of the counts. The zooplankton origin difference in Exp.1 led to no differences in the common taxa identified in the tanks.

## Hydra in one experiment

In Exp. 1, there were *Hydra* in all tanks. *Hydra* are small invertebrates that attach to surfaces and prey on zooplankton. Evidently we did not remove them all from the collected zooplankton before adding the zooplankton to the tanks (see above in Methods), and we expect that when we mixed zooplankton in the tanks via redistribution, that this distributed *Hydra* as well, leading to their presence in all but three tanks. Those three tanks were excluded from the experiment.

### Table S1. Minor differences in design and implementation of experiments.

| Exp. | # Replicates no-fish treatment | # Replicates fish treatment | # fish | Fish length (cm, mean +/- stdev) | N:P ratio | Mesh size (µm) of sieve for zoo- plankton collection from tanks |
| --- | --- | --- | --- | --- | --- | --- |
| 1 | 12* | 6 | 3 | 4.7 ± 0.4 | 15:1 | 53 |
| 2 | 16* | 8 | 2 | 5.9 ± 0.4 | 15:1 | 53 |
| 3 | 9 | 9 | 2 | 6.4 ± 0.3 | 20:1 | 64 |
| 4 | 14 | 14 | 2 | 5.8 ± 0.6 | 20:1 | 64 |

| Exp. | Day nutrient & phyto-plankton pulse added | Day zoo-plankton added | Day zoo- plankton mixed | Date treatments initiated | Day of zoo- plankton sampling |
| --- | --- | --- | --- | --- | --- |
| 1 | -45 | -41 to -42 | -20 | 7/14/03 | 59 |
| 2 | -35 | -27 | -13 | 7/6/06 | 71 |
| 3 | -56 | -49 & -38 | -24 & -6 | 7/8/13 | 44 |
| 4 | -38 | -32 | -14 & -7 | 7/14/14 | 31 |

* Includes invertebrate treatment

# Supporting Information B: Statistics of particular experimental treatments

For Exps. 1 and 2, the difference between invertebrate and control treatments in the zooplantion community was evaluated using PERMANOVAs and for individual taxa using generalized linear models based on quasi-Poisson distribution. Results indicated no significant differences in the multivariate response (Experiment 1: *F*_1,12_ = 1.27, *p* = 0.27; Experiment 2: *F*_1,14_ = 1.38, *p* = 0.22;) or in the individual taxa (Table S1).

For Exp. 4, the effect of periphyton removal and the interaction between periphyton removal and fish kairomone was evaluated for the zooplankton community using a PERMANOVA and for individual taxa using generalized linear models based on quasi-Poisson distribution. Results indicated no significant differences in the multivariate response (periphyton effect: *F*_1,25_ = 0.65, *p* = 0.68; interaction: *F*_1,23_ = 1.30, *p* = 0.26) or in the individual taxa, except for cyclopoid copepods (Table S2).

Table S2. P-values associated with difference between control and invertebrate treatments in Exps. 1 and 2, and the effect of periphyton removal and the interaction between periphyton removal and fish kairomone for Exp. 4. Species listed in same order as Table S1. Species absent or very low densities indicated by not applicable (na).

|  | Exp. 1 | Exp. 2 | Exp. 4 | |
| --- | --- | --- | --- | --- |
| Taxa | Main | Main | Main | Interaction |
| *Ceriodaphnia* | 0.39 | 0.98 | 0.71 | 0.08 |
| *Bosmina* | 0.62 | 0.22 | 0.23 | 0.07 |
| *D.pulicaria* | 0.92 | 0.47 | 0.26 | 0.57 |
| *Alona* | 0.87 | 0.3 | 0.76 | 0.48 |
| *Chydorus* | 0.16 | 0.31 | 0.67 | 0.89 |
| *Diaphanosoma* | 0.67 | 0.31 | 0.33 | 0.19 |
| Ostracod | 0.8 | 0.84 | 0.82 | 0.75 |
| Cyclopoid | 0.18 | 0.85 | 0.01 | 0.69 |
| Calanoid | 0.67 | 0.17 | 0.69 | 0.78 |
| *Scapholeberis* | 0.48 | 0.72 | 0.74 | 0.05 |
| *D.dentifera* | na | 0.82 | 0.83 | 0.37 |
| *Eurycerus* | 1 | 0.54 | na | na |
| *Simocephalus* | 0.16 | 0.18 | na | na |
| *D.parvula* | 0.52 | na | na | na |
| *Pleuroxus sp.* | na | 0.31 | 0.19 | 1 |

# Supporting Information C: Nutrient addition schedule

Nutrients in the form of NH_4_NO_3_ and KH_2_PO_4_ were added to mesocosms to support phytoplankton growth at a N:P ratio of 15:1 in Exps. 1 and 2, and 20:1 in Exps. 3 and 4. An initial pulse was added concurrent with adding phytoplankton to the tanks, and thereafter weekly maintenance was added (Fig. C1). The initial pulse of KH_2_PO_4_ was 0.37g in Exps. 1, 3 and 4, and 0.57 g in Exp. 2. In Exps. 1, 3 and 4, nutrients were mixed in water in 5 gallon buckets and then added to tanks 2 times (Exp. 1) or 5 times (Exps. 2 and 3) per week. In Exp. 2, a peristaltic pump was used to deliver nutrients continuously.

Fig. C1. Cumulative mass (g) of KH_2_PO_4_ added to tanks in the four experiments.

# Supporting Information D. Power analysis or Exp. 3 analysis versus other treatments.

The lack of an effect on any species in Exp. 3 could be due to two sources: low sample size or low effect size, each of which would lead to low power to detect any differences that were there. However, sample sizes for Exp. 3 were actually larger for many species than in experiments where significant effects of fish were detected (Table S1). A comparison of effect sizes (calculated as $\left| \bar{\mu_{C}}-\bar{\mu_{F}} \right|/\sigma$, where $\bar{\mu_{C}}$ is the mean density in the control treatment, $\bar{\mu_{F}}$ is the mean density in the fish treatment, and $\sigma$ is the shared standard deviation in density across treatments) indicates that effect sizes were lower in Exp. 3 for all taxa than in any experiment where significant effects were observed. This lower effect size could be due to a lower difference between the means (indicating contingency in the effect of predators) or due to higher variability. A comparison of $\bar{\mu_{C}}/\sigma$ values across experiments indicates that, in many cases, Exp. 3 was less variable than experiments where significant results were observed, indicating that the lack of significant effects in Exp. 3 was truly because predators did not affect zooplankton density.

# Supporting Information E. NMDS Plots

# Fig. E1: Scatterplots showing the distribution of zooplankton communities along two NMDS axes. Ellipses show how communities sort based on experiment and treatment.

#
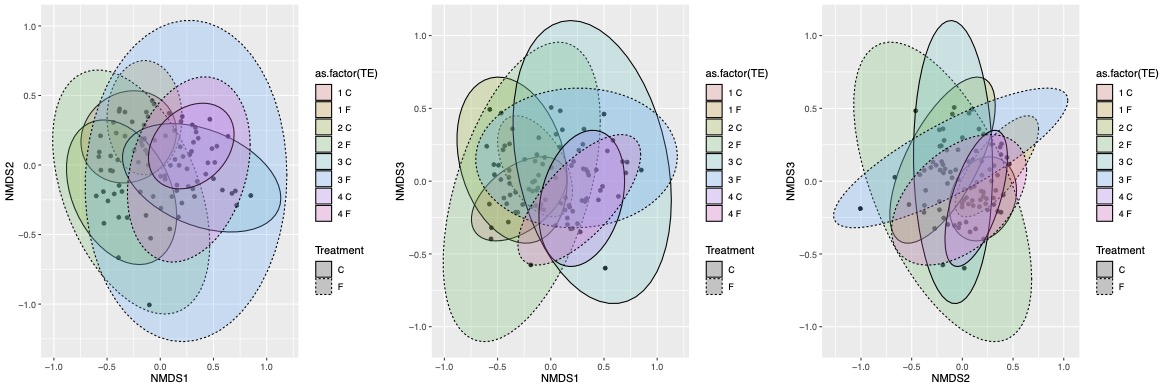

Supplement: Supplementary file 1 — Data S1. [file ECE3-15-e70861-s001.docx]
